# Platelet-rich plasma-derived exosomes establishing a muscular proregenerative microenvironment through enhancing the viability of fibro-adipogenic progenitors

**DOI:** 10.1038/s12276-025-01606-x

**Published:** 2025-12-25

**Authors:** Xin Ma, Jin Qian, Jia Cai, Yu-xin Wang, Wei Li, Xiao-yan Zhu, Ri-zhao Pang, Hui-zhen Zou, Meng-meng Yang, Li-ping Liu, Mu Yuan, Gao-ming Li, Lin-jie Wang, Yi Yang, Ji-wu Chen, Zhu Huang, Xiao-wei Qi, Xia Kang

**Affiliations:** 1 Tissue Stress Injury and Functional Repair Key Laboratory of Sichuan Province and Basic Medical Laboratory, General hospital of Western Theater Command, Chengdu, China; 2 Rehabilitation Medicine department, The General Hospital of the Western Theater Command and Sichuan Provincial Clinical Medical Research Center for Traditional Chinese Medicine Orthopedics and Sports Rehabilitation, Chengdu, China; 3 College of Medicine, Southwest Jiaotong University, Chengdu, China; 4 Center for Medical Epigenetics, School of Basic Medical Sciences, Chongqing Medical University, Chongqing, China; 5 Department of Transfusion Medicine, The General Hospital of Western Theater Command, Chengdu, China; 6 Hyperbaric Oxygen Therapy Center, The General Hospital of Western Theater Command, Chengdu, China; 7 Disease Surveillance Division, Center for Disease Control and Prevention of the Central Theater Command, Beijing, China; 8 Department of Sports Medicine, Shanghai General Hospital, Shanghai Jiaotong University, Shanghai, China; 9 Breast and Thyroid Surgery, Southwest Hospital, Army Medical University, Chongqing, China

**Keywords:** Stem-cell differentiation, Mesenchymal stem cells, Cell signalling

## Abstract

Clinical studies have shown a paradox of the usage of platelet-rich plasma (PRP) on treating fatty infiltration (FI) in injured muscles. However, the underlying reason is still unclear, partially owing to unknown effective components and confounders. Here we found that exosomes derived from PRP (thereafter named PRP-exos) most efficiently prevented FI in injured muscles by inhibiting the adipogenesis of fibro-adipogenic progenitors (FAPs). Importantly, we found aging largely impaired the therapeutic effects of PRP-exos. Mechanistically, miRNA cargoes in PRP-exos mediated the effects of PRP-exos on adipogenesis of FAPs as well as FI in injured muscles, of which, hsa-let-7f-5p and hsa-miR-16-5p were the two most important components. TGFBR3 was identified as a new cotarget gene of these two miRNAs and a new regulator to control the adipogenesis of FAPs. The FI in muscles can be significantly reduced after conditional knockout of TGFBR3 in FAPs. In addition, we further investigated that TGFBR3 regulated the activation of ERK–PPARγ pathway through directly inducing the degradation of KRT10, and thus impacted the adipogenesis of FAPs. Interestingly, PRP-exos or these two miRNAs can preserve the viability and promote the proregenerative supporting capacity of FAPs by targeting TGFBR3 to facilitate muscle regeneration. Collectively, our findings identified the effective components in PRP to inhibit FI and support muscle regeneration. Furthermore, the negative influence of aging on clinical applications of PRP cannot be neglected.

## Aging impairs PRP exosome efficacy in muscle injury recovery

Platelet-rich plasma (PRP) is often used to help heal injuries, especially in muscles. Here researchers studied PRP to find out more about its effects on muscle healing. They focused on exosomes in PRP from young and old donors. They found that exosomes from young donors were better at preventing fat buildup in injured muscles. This is important because fat buildup can slow down healing. The study involved human blood samples and experiments on mice to see how these exosomes affected muscle cells. Researchers discovered that certain molecules in the exosomes, called miRNAs, were key to their healing effects. These miRNAs targeted a gene called TGFBR3, which plays a role in cell growth and fat formation. By understanding this process, the researchers hope to improve treatments for muscle injuries.

This summary was initially drafted using artificial intelligence, then revised and fact-checked by the author.

## Introduction

Platelet-rich plasma (PRP) has been widely used in the treatment of a variety of diseases. It is well known that administration of PRP could promote tissue regeneration and repair^1–5^. As the dominant organ to produce motion, muscle is soft and superficial, which is susceptible to injury. Some studies reported to PRP can be an ideal treatment to efficiently promote the muscle regeneration after injury^3,6^, however, it remains elusive that the underlying mechanisms and target cell types that PRP exerts its therapeutic effects on muscle injury.

Although the activation of muscle stem cells (MuSCs) is a critical event to muscle regeneration, microenvironment remodeling in stem cell niche determines the fate of MuSCs and the final outcomes. Fatty infiltration (FI) is one of the most important pathological changes in injured muscles^7,8^. Accumulation of adipocytes gradually replaces functional muscle fibers, leading to impaired muscle quality and unfavorable muscle recovery. In some cases, such as rotator cuff tears (RCT), FI can even cause surgery failure. A clinical study revealed that injection of PRP could attenuate FI in moderate-to-large RCTs, however, another study found that PRP treatment cannot improve the severity of FI in small-to-medium-sized RCTs^1,9^. The underlying reasons leading to this paradox remain elusive and should be further clarified. In addition, several potential confounding factors in using PRP therapy cannot be neglected. Most importantly, as PRP is a product of autologous blood, it is unclear whether intrinsic heterogeneity, such as aging, could have unpredictable therapeutic effects since some studies proved that aging could significantly alter the components of blood. Determining the key component is helpful to clarify the underlying mechanisms for the unstable therapeutic effects of PRP^6,10^.

Fibro-adipogenic progenitors (FAPs) are the primary cellular source of FI in muscle^11–14^. The association between FAPs and the severity of FI is highly correlated with the number of FAPs and the adipogenic capacity of FAPs^15^. Numerous studies have proven that adipogenesis of FAPs can occur under a range of conditions both in vivo and in vitro. In vitro, insulin was thought to be the most important regulator to induce adipogenesis of FAPs^11^. The adipogenic capacity of FAPs could be enhanced in a hyperlipidemic environment and can be inhibited by the upregulation of IL-4/IL-13, myokines, the hedgehog pathway and so on^13–17^. Moreover, the accumulation of FAPs significantly impacts the severity of FI as demonstrated by us and others musclin, TNF and other cytokines can reduce FI through regulating the proliferation and apoptosis of FAPs^15,18–20^. On the other hand, since the activation of FAPs is essential to muscle regeneration, it is ideal that the adipogenic capacity of FAPs is inhibited while the activation of FAPs is preserved^13,16^. The fate of FAPs, such as differentiation and proliferation, is regulated by microenvironment changes^14–16,21,22^. Considering this, it is interesting to know how and which bioactive factors in PRP regulate the cellular fate of FAPs.

In this study, we found that exosomes in PRP from young donors (hereafter referred to as Y-PRP-exos), which are enriched in hsa-let-7f-5p and hsa-miR-16-5p, serve as key regulators in preventing both FAP adipogenesis and FI—an effect not observed in PRP exosomes from old donors (hereafter referred to as O-PRP-exos). TGFBR3 was the common target gene of these two miRNAs. Downregulation or conditional knocked out of TGFBR3 in FAPs prevented FI through regulating the KRT10–ERK–PPARγ pathway. We also found that Y-PRP-exos preserved the proregenerative capacity of FAPs and the secreta from FAPs treated by Y-PRP-exos promoted the activation of MuSCs and muscle regeneration through upregulation of IGFBP6. Our study demonstrates the importance of PRP-exos in mediating the PRP-exerted effects on FI or muscle regeneration. hsa-let-7f-5p, hsa-miR-16-5p and their downstream, TGFBR3, could be a new potential therapeutic targets in muscle recovery.

## Methods

### Human samples and preparation of PRP exosomes

Human blood samples were collected from five young men and five old men. The average age of young donors and old donors were 22.0 and 66.6 years, respectively. All participants provided informed consent, and the procedure was approved by the Ethics Committee of General Hospital of Western Theater Command (approval no. 2023EC2-ky002).

For the preparation of PRP, a regular blood draw was performed to collect donor blood. Fifty milliliters of venous blood was extracted from each donor and stored. Blood was centrifuged at 580*g* for 8 min at room temperature. After centrifugation, the plasma fraction was collected in a tube.

### Extraction and identification of PRP-derived exosomes

PRP was centrifuged at 2,000*g* for 30 min and 10,000*g* for 1 h to remove dead cells and debris. Then, the supernatant was filtered through a 0.22-μm filter. The soluble factors (SF) were collected after 1 h and 20 min of centrifugation at 100,000*g*. The pellet was resuspended in 15 ml of precooled PBS and centrifuged at 100,000*g* for 70 min. Then, the final pellet (exosomes) was resuspended in precooled PBS. The concentration and size distribution of the exosomes were measured using a ZetaView S/N 17-310 (Partical Metrix, GmbH). Transmission electron microscopy was used to assess exosome morphology. The levels of exosomal surface marker proteins, including TSG101, calnexin, CD9 and CD63, were analyzed by western blotting.

### Animal experiments

All procedures were approved by the Ethics Committee of General Hospital of Western Theater Command (approval no. 2022EC3-ky065). C57BL/6 mice at 6–8-weeks old were purchased from Hunan SJA Laboratory Animal Co., Ltd. The mice were housed under a 12-h light/dark cycle and fed a standard laboratory diet and water ad libitum.

FI in injured muscles was induced by intramuscular injection of glycerol following previously published protocols^15^. Briefly, the mice were anesthetized and the hair on the hind limb was carefully removed. After sterilization with 75% alcohol, 50 μl (v/v) of glycerol was injected into the muscle belly of the tibias anterior muscles. In some experiments, 1.0 × 10^9^ exosomes isolated from PRP of young donors (hereafter named Y-PRP-exos) or old donors (hereafter named O-PRP-exos), 5 μg scRNA or miRNAs were mixed with transfection reagent and then were injected into tibias anterior muscles twice.

### Cell preparation and FACS

Primary FAPs were isolated as described. Briefly, muscles from both hind limbs were collected and minced. Then the tissue was digested with 0.2% type Ⅱ collagenase (Worthington) for 1 h at 37 °C. Muscle slurries were filtered through 100-μm and 40-μm cell strainers (BD Bioscience). After the erythrocytes were eliminated, the cells were resuspended in washing buffer consisting of PBS containing 2% FBS. The cells were stained with antibodies for 30 min at 4 °C in the dark. Then, the stained cells were isolated by a FACSAria III system (BD Biosciences). Information on the antibodies is listed in Supplementary Table 1.

### Cell culture

FAPs were cultured in growth medium consisting of Dulbecco’s modified Eagle medium (DMEM) with 20% FBS and 2% penicillin‒streptomycin. The medium was changed every 3 days. For inducing adipogenesis, FAPs were exposed to adipogenic induction medium consisting of DMEM with 20% FBS, 0.5 mM IBMX (Sigma-Aldrich), 0.25 μM dexamethasone (Sigma-Aldrich) and 10 μg/ml insulin (Procell) for up to 3 days, and adipogenic maintenance medium consisting of DMEM with 10% FBS and 10 μg/ml insulin. In some experiments, 1.0 × 10^8^ exosomes or 2 μg miRNAs were added to the culture medium.

### Histochemistry and cytochemistry

Immunofluorescence or cytochemistry followed previous protocols^15^. Briefly, fresh frozen muscle tissues were sectioned into 8-μm sections. The sections were collected using isopentane methods. The sections were blocked for 1 h at 37 °C in PBS containing 10% normal donkey serum, 3% BSA and 0.1% Triton X-100 and incubated with primary antibodies at 4 °C overnight. After excessive primary antibodies were washed away, the sections were cultured in secondary antibodies conjugated to AF488, Cy3 or phalloidin conjugated to AF647 or TRITC. Then the sections were counterstained with Hoechst 33342 or DAPI (Beyotime). The primary and secondary antibodies used are listed in Supplementary Table 1. For Oil Red O staining, cells were fixed in 4% PFA for 10 min and rinsed in water followed by 60% isopropanol. Then the samples were stained in Oil Red O for 15 min and rinsed in water. Cells were visualized using an IX81 fluorescence microscope (Olympus) equipped with a CCD camera. Confocal images of muscle sections were captured using the confocal laser scanning microscope system TCS SP8 (Leica Microsystems) or Nikon A1R HD25 (Nikon).

### Real-time PCR

Total RNA in cells was extracted using TRIzol reagent. RNA was reverse transcribed into cDNA by using a PrimeScriptRT Master Mix kit (TAKARA) following the manufacturer’s protocol. The mRNA levels of the target genes were normalized to the mRNA levels of GAPDH. Relative mRNA expression was calculated using the 2−ΔΔ*Cq* method. The primer sequences are listed in Supplementary Table 1.

### Dual-luciferase reporter system

The 293 T cells were seeded into 96-well plates. Cells were transfected with m-tgfbr3-3′ UTR and 5 pmol hsa-miR-16-5p, hsa-let-7f-5p or negative control following the manufacturer’s instructions (Hanbio). Luciferase assays were performed with the dual-luciferase reporter assay system (Hanbio) according to the manufacturer’s instructions. Luminescent signals were quantified by a luminometer.

### Western blotting

Total proteins (20 μg) were separated using SDS–PAGE gels. The proteins were transferred to polyvinylidene difluoride membranes and then blocked with 5% (w/v) skim milk diluted in TBST for 1 h at room temperature. Then, the membranes were incubated with primary antibodies at 4 °C overnight. After the specimens were washed with TBS, secondary antibodies were added and incubated at room temperature for 2 h. Densitometric analysis was performed using a ChemiDoc Touch Imaging System (Bio-Rad Laboratories). The primary and secondary antibodies used are listed in Supplementary Table 1.

### RNA-seq or miRNA-seq

For RNA-sequencing (RNA-seq), total RNA was extracted using TRIzol reagent (Invitrogen). Sequencing libraries were prepared using the KC-Digital Stranded mRNA Library Prep Kit for Illumina (Wuhan Seqhealth Co.) following the manufacturer’s protocols. Sequencing was performed on an Illumina NovaSeq platform (Illumina). Trimmed sequence reads were mapped to the mouse genome (mm10) using STAR software (version 2.5.3a), and read count quantification was performed using FeatureCounts (version 1.5.1). The significantly differentially expressed genes (DEGs) identified from the comparative transcriptomic analysis were utilized for downstream functional enrichment analyses, including Gene Ontology (GO) enrichment, Kyoto Encyclopedia of Genes and Genomes (KEGG) pathway enrichment and Gene Set Enrichment Analysis (GSEA), using the clusterProfiler R package (version 4.4.4). GO enrichment analysis was performed based on gene annotations provided by the org.Mm.eg.db database (corresponding to *Mus musculus*), while KEGG pathway enrichment utilized data from the KEGG.db and KEGGREST databases. For GO and KEGG enrichment, the hypergeometric test (overrepresentation analysis, ORA) was applied to evaluate whether specific biological terms or pathways were statistically overrepresented in the DEG list. For GSEA, genes were preranked according to their log_2_ fold change, and enrichment scores were calculated based on a permutation-based algorithm to assess whether predefined gene sets were significantly enriched at the top or bottom of the ranked list. Unless explicitly stated, all parameters were used with their default settings as provided in the clusterProfiler package. For miRNA-seq, total RNA was extracted from Y-PRP-exos or O-PRP-exos using an exoRNeasy Midi Kit (Qiagen). Sequencing libraries were generated using a QIAseq miRNA Library Kit (Qiagen) for Illumina according to the manufacturer’s instructions. Sequencing was performed on an Illumina Nova seq platform (Illumina). The experiments and analysis were performed by Wayen Biotechnologies.

### BrdU assay

BrdU assays were performed using a Phase-Flow BrdU Kit (BioLegend) following the manufacturer’s protocol. Briefly, for the in vivo assay, the mice were injected with BrdU solution (10 mg/ml) at 12 h and 3 h before collection. The cell suspension was prepared and incubated with antibodies against surface markers following the protocol used for FACS. After excessive antibodies were washed away, the cell suspension was fixed, permeabilized and treated with DNase for 1 h at 37 °C. Then, the samples were incubated with a BrdU antibody conjugated to Phycoerythrin (PE) for 30 min. For the in vitro assay, the cells were treated with BrdU solution for 3 h before collection and the samples were then treated following the same procedures as in vivo. The specimens were detected by flow cytometry (Beckman Coulter). The data were analyzed by using FlowJo v10 (FlowJo, LLC.). Information about the antibodies is provided in Supplementary Table 1.

### Statistical analysis

The results are shown as the mean ± s.d. A Student’s *t*-test was used to compare two groups. For more than two groups, a one-way analysis of variance (ANOVA) was used. Statistical significance was considered at *P* < 0.05. All experiments were repeated at least three times.

## Results

### Y-PRP-exos acting as the key regulator for inhibiting the adipogenesis of FAPs and preventing FI in injured muscles

We first that confirmed treatment of PRP collected from young donors (Y-PRP) can significantly inhibit the adipogenesis of FAPs in vitro (Supplementary Fig. 1a). Then, SF and exosomes were isolated through gradient centrifugation and used to treat FAPs, respectively (Supplementary Fig. 1b). The results indicated that PRP-exos collected from young donors (Y-PRP-exos) were more efficient at inhibiting adipogenesis of FAPs than SF collected from young donors (Y-SF) (Supplementary Fig. 1c), confirming that exosomes play a central role in mediating PRP’s suppression of FAP adipogenesis.

**Fig. 1:**
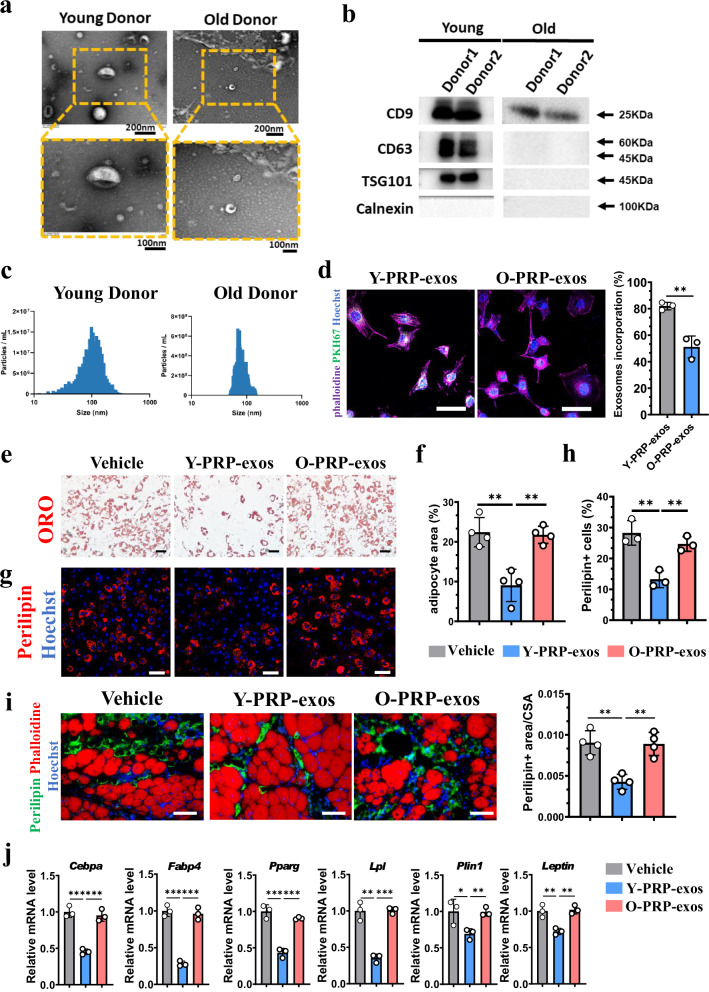
Y-PRP-exos inhibit adipogenesis of FAPs and FI in injured muscles. **a** PRP-exos were isolated from two young donors and two old donors. Representative images of the appearance of exosomes detected by electron microscopy (top; scale bar, 200 nm) and enlarged view (bottom; scale bar, 100 nm). **b** Western blot analysis detecting the protein expression of several classic biomarkers in Y-PRP-exos and O-PRP-exos. **c** NTA analysis detecting the size distribution of Y-PRP-exos and O-PRP-exos. **d** Fluorescence detection of the PKH67-labeled Y-PRP-exos or O-PRP-exos that were absorbed by FAPs in vitro (left) and quantification of the percentage of PKH67-positive exosomes in FAPs (right). **e**, **f** Representative images of adipogenic induction of FAPs after treatment of Y-PRP-exos or O-PRP-exos detected by Oil red O staining (ORO) (scale bar, 50 μm) (**e**) and quantification of the area of adipocytes in each group (**f**). *n* = 4 per condition. **g**, **h** Representative images of immunofluorescence (**g**) and quantification (**h**) of perilipin-positive FAPs after being induced by adipogenic induction medium followed by treatment of Y-PRP-exos or O-PRP-exos (scale bar, 100 μm). *n* = 3 per condition. **i** Representative images of the accumulation of adipocytes in glycerol-induced injured muscles at 14 DPI after treatment of Y-PRP-exos or O-PRP-exos (left) (scale bar, 100 μm) and quantification of FI area (right). *n* = 4 per condition. **j** RT–PCR detecting the mRNA levels of adipogenic biomarkers in primary FAPs isolated from glycerol-injured muscles at 7 DPI. *n* = 3 per condition.

Since aging could be the most common confounder to interfere with the effects of blood products, we isolated the PRP-exos from young donors (Y-PRP-exos) and old donors (O-PRP-exos). The successful isolation of exosomes from PRP was confirmed by electronic microscopy (Fig. 1a). For surface biomarkers, proteins were extracted from the same number of Y-PRP-exos and O-PRP-exos. Calnexin was not detected in PRP-exos from either young or old donors. Furthermore, three classic biomarkers, CD9, CD63 and TSG101, were highly expressed in Y-PRP-exos, but the protein levels of CD63 and TSG101 were extremely low in O-PRP-exos (Fig. 1b). Nanoparticle tracking analysis (NTA) showed that the diameter distribution of Y-PRP-exos was broader than that of O-PRP-exos (Fig. 1c). Notably, the rate of O-PRP-exos absorbed in FAPs was significantly lower than that in Y-PRP-exos (Fig. 1d). These results demonstrate that PRP-exos from young donors and old donors exhibit obviously distinct characteristics.

Next, we detected whether differences could also be found in the effects of Y-PRP-exos or O-PRP-exos on the adipogenesis of FAPs and FI in muscles. Oil Red O staining showed that adipogenesis of FAPs was significantly inhibited by treatment of the Y-PRP-exos compared to the O-PRP-exos or control groups (Fig. 1e, f). Similarly, the percentage of perilipin-positive FAPs was lowest after treatment of Y-PRP-exos compared to the control or O-PRP-exos groups (Fig. 1g, h). Consistently, FI was markedly attenuated in the Y-PRP-exos treated group compared to the other two groups (Fig. 1i). The expression of classic biomarkers of adipogenesis in FAPs freshly isolated from injured muscle at 7 days post injury (DPI) was significantly downregulated in the Y-PRP-exos treated group, but not in the O-PRP-exos treated group (Fig. 1j). Interestingly, we also found the fibrosis was enhanced in vivo after treatment of Y-PRP-exos compared to the control group (Supplementary Fig. 1d). In addition, treatment of Y-PRP-exos showed little toxicity on major organs (Supplementary Fig. 1e). These data indicated that Y-PRP-exos directly inhibited the adipogenesis of FAPs and prevented FI in injured muscles.

### Y-PRP-exos preserve the viability of FAPs

**Fig. 2:**
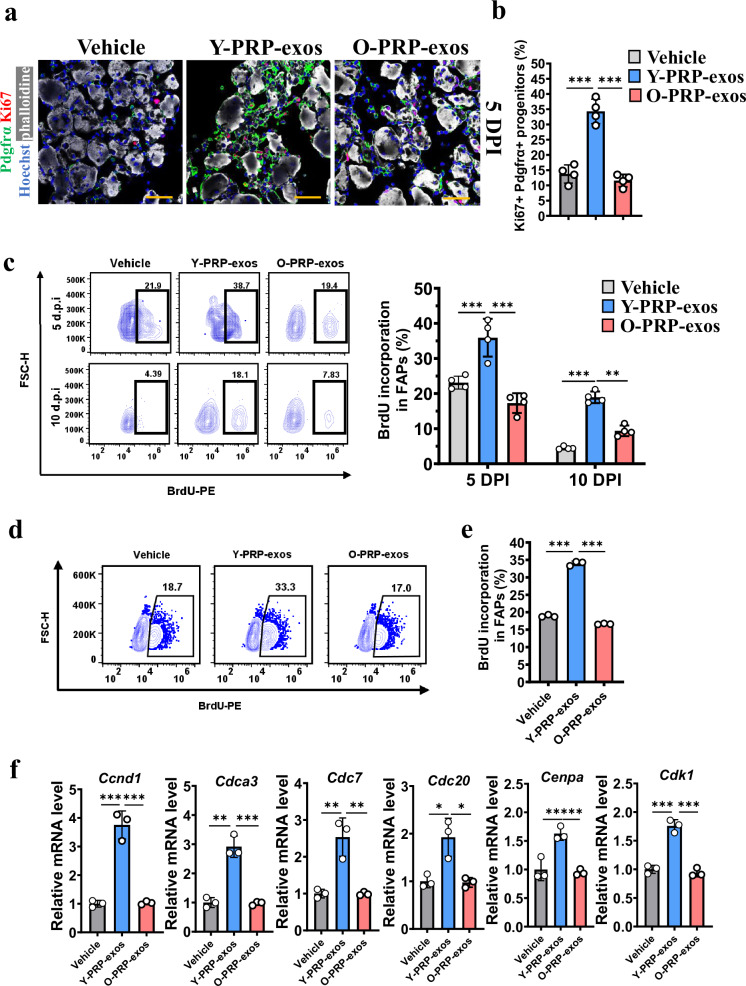
Y-PRP-exos promote the proliferation of FAPs in vitro and in vivo. **a**, **b** Representative images of Ki67-positive FAPs in glycerol-induced injured muscles at 5 DPI after treatment of Y-PRP-exos or O-PRP-exos (scale bar, 50 μm) (**a**) and quantification of Ki67-positive FAPs (**b**). *n* = 4 per condition. **c** Flow cytometry plots (left) and quantification (right) of BrdU incorporation in FAPs after treatment with Y-PRP-exos or O-PRP-exos at 5 DPI and 10 DPI. *n* = 4 per condition. **d**, **e** Flow cytometry plots (**d**) and quantification (**e**) of BrdU incorporation in FAPs after treatment of Y-PRP-exos or O-PRP-exos for 48 h in vitro. *n* = 3 per condition. **f** RT–PCR detecting the mRNA levels of cell cycle biomarkers in FAPs after treatment of Y-PRP-exos or O-PRP-exos for 48 h in vitro. *n* = 3 per condition.

Since the inhibition of FI could also be impacted by the number of FAPs, we next detected how PRP-exos regulates the proliferation of FAPs. The results showed that treatment of Y-PRP-exos, but not O-PRP-exos, significantly upregulated the Ki67-positive FAPs in injured muscles (Fig. 2a, b). Consistently, BrdU incorporation was also upregulated in FAPs after administration of Y-PRP-exos at 5 DPI or 10 DPI compared to in the O-PRP-exos or control groups (Fig. 2c). To confirm that Y-PRP-exos directly regulates the proliferation of FAPs, Y-PRP-exos or O-PRP-exos were added to stimulate FAPs in vitro. The results showed that the proliferation rate was upregulated in the Y-PRP-exos treated group compared to the other two groups (Fig. 2d, e). The mRNA levels of cell cycle biomarkers were also upregulated after treatment of Y-PRP-exos (Fig. 2f). These data indicate that Y-PRP-exos preserve the viability of FAPs while inhibiting adipogenesis.

### miRNA cargoes mediate the effects of Y-PRP-exos on regulating the cellular fate of FAPs

As various bioactive components are contained in exosomes, we removed nucleic acids or proteins to determine the most critical component that mediates the effects of Y-PRP-exos on regulating the cellular fate of FAPs (Supplementary Fig. 2a, b). Interestingly, we found that depletion of RNAs markedly reversed the effects of Y-PRP-exos on inhibiting the adipogenesis of FAPs (Supplementary Fig. 2c, d). Similarly, the proliferation of FAPs were barely impacted by treatment of Y-PRP-exos without RNAs (Supplementary Fig. 2e). These results demonstrate that RNAs could be the key regulator in Y-PRP-exos to regulate the fate of FAPs.

**Fig. 3:**
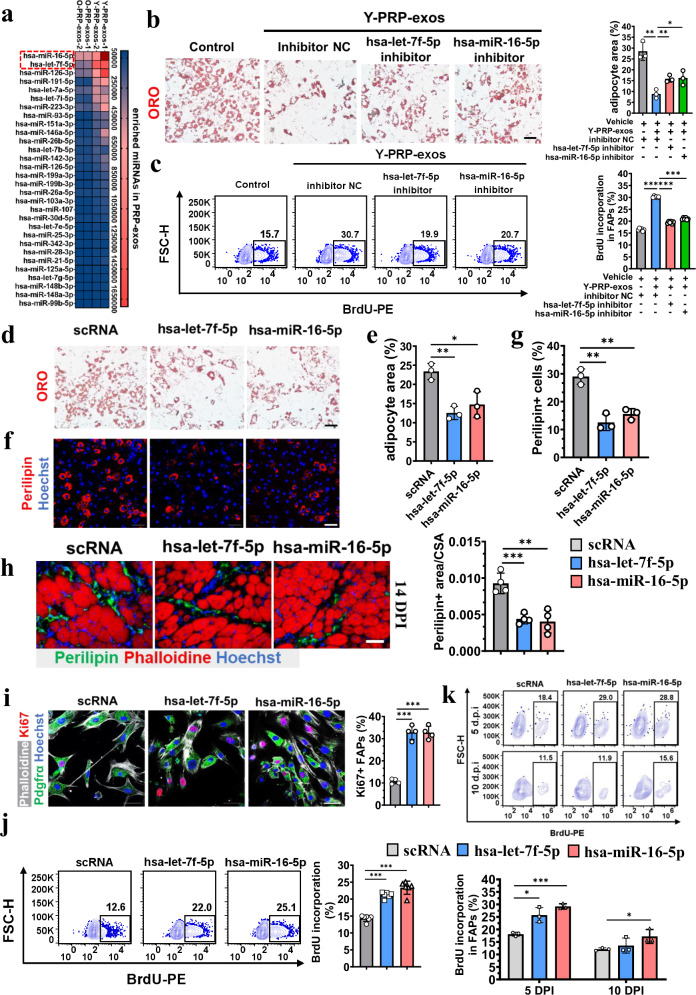
hsa-let-7f-5p and hsa-miR-16-5p mediate the effects of Y-PRP-exos on adipogenesis and proliferation of FAPs. **a** A heat map of the top 30 miRNAs in Y-PRP-exos and O-PRP-exos. **b** Representative images of adipogenic induction of FAPs after treatment of Y-PRP-exos with or without transfection of hsa-let-7f-5p inhibitor or hsa-miR-16-5p inhibitor detected by Oil red O (ORO) (left; scale bar, 50 μm) and quantification of the area of adipocytes in each group (right). *n* = 3 per condition. **c** Flow cytometry plots (left) and quantification (right) of BrdU incorporation in FAPs after treatment of Y-PRP-exos with or without hsa-let-7f-5p inhibitor or hsa-miR-16-5p inhibitor in vitro. *n* = 4 per condition. **d**, **e** Representative images of adipogenic induction of FAPs after transfection with hsa-let-7f-5p and hsa-miR-16-5p (scale bar, 50 μm) (**d**) and quantification of the area of adipocytes in each group (**e**). *n* = 3 per condition. **f**, **g** Representative images of immunofluorescence (**f**) and quantification (**g**) of perilipin-positive FAPs after transfection with hsa-let-7f-5p and hsa-miR-16-5p (scale bar, 100 μm). *n* = 3 per condition. **h** Representative images of FI in glycerol-induced injured muscles at 14 DPI after injection of hsa-let-7f-5p or hsa-miR-16-5p (scale bar, 100 μm) (left) and quantification of FI area (right). *n* = 4 per condition. **i** Representative images of immunofluorescence (left) and quantification (right) of Ki67-positive FAPs after transfection with hsa-let-7f-5p and hsa-miR-16-5p (scale bar, 50 μm). *n* = 3 per condition. **j** Flow cytometry plots (left) and quantification (right) of BrdU incorporation in FAPs after transection of hsa-let-7f-5p or hsa-miR-16-5p in vitro. *n* = 6 per condition. **k** Flow cytometry plots (left) and quantification (right) of BrdU incorporation in FAPs after injection of hsa-let-7f-5p or hsa-miR-16-5p in glycerol-induced injured muscle at 5 DPI and 10 DPI. *n* = 3 per condition.

Considering that PRP-exos have cross-species effects, we speculated that miRNAs may be important in mediating the effects of Y-PRP-exos on FAPs, since human-derived miRNAs have been demonstrated to regulate the cellular behavior of other species^23^. miRNA-seq showed that most miRNAs (38) could be identified in both Y-PRP-exos and O-PRP-exos (Supplementary Fig. 2f). In addition, hsa-let-7f-5p, hsa-miR-16-5p and hsa-miR-126-3p were the top three enriched miRNAs in PRP-exos of all donors (Supplementary Fig. 2g). These results indicated that the heterogeneity of miRNAs in PRP-exos was low, regardless of age. However, the abundance of miRNAs in Y-PRP-exos and O-PRP-exos was quite different, especially in high-ranking miRNAs (Fig. 3a). The expression of hsa-miR-16-5p and hsa-let-7f-5p in O-PRP-exos from two old donors was approximately one-quarter to one-half of that in Y-PRP-exos from the two young donors (Table 1), which may partially explain why O-PRP-exos had little effect on regulating the fate of FAPs.

**Table 1:** Top five enriched miRNAs in PRP-exos derived from young donors or old donors.

	Ranking	PRP-exos-1			PRP-exos-2		
		miRNA	Reads	Percentage of total	miRNA	Reads	Percentage of total
Young	1	hsa-miR-16-5p	1,788,024	16.15701078	hsa-let-7f-5p	1,383,348	19.25052
	2	hsa-miR-126-3p	1,685,697	15.23235964	hsa-miR-16-5p	875,493	12.18327
	3	hsa-let-7f-5p	1,439,648	13.00900226	hsa-miR-126-3p	699,988	9.740958
	4	hsa-miR-191-5p	680,555	6.149657093	hsa-miR-191-5p	490,885	6.831103
	5	hsa-miR-223-3p	669,926	6.053610917	hsa-let-7a-5p	482,076	6.708518
Elder	1	hsa-miR-16-5p	507,219	18.0171697	hsa-miR-16-5p	504,548	17.6421245
	2	hsa-let-7f-5p	327,267	11.6250083	hsa-let-7f-5p	300,878	10.5205592
	3	hsa-miR-126-3p	296,063	10.516596	hsa-miR-126-3p	283,914	9.92739269
	4	hsa-let-7a-5p	207,568	7.3731226	hsa-let-7a-5p	200,267	7.00257526
	5	hsa-miR-223-3p	157,560	5.59676442	hsa-miR-223-3p	194,852	6.81323331

We next explored the importance of hsa-miR-16-5p and hsa-let-7f-5p in the effects of Y-PRP-exos on the adipogenesis and proliferation of FAPs. The results showed that the adipogenesis and proliferation of FAPs regulated by Y-PRP-exos can be reversed after transfection with these two miRNAs (Fig. 3b, c). Oil red O staining showed that the number of adipocytes was significantly reduced after transfection with these two miRNAs (Fig. 3d, e). Considering that these two miRNAs may possibly impact the number of FAPs, we also calculated the percentage of perilipin-positive cells in total cells. Consistently, we found that the percentage of perilipin-positive cells after transfection with hsa-let-7f-5p or hsa-miR-16-5p was also significantly lower than in the control group (Fig. 3f, g). These results indicate that hsa-let-7f-5p and hsa-miR-16-5p can directly inhibit adipogenesis of FAPs in vitro. After treatment with exogenous hsa-let-7f-5p or hsa-miR-16-5p, FI in glycerol-injured muscles was significantly prevented (Fig. 3h).

Next, we detected the effects of these two miRNAs on the proliferation of FAPs. The percentage of Ki67-positive FAPs was significantly higher in the miRNA-treated group than in the control group (Fig. 3i). Consistently, flow cytometry analysis indicated that BrdU incorporation in FAPs was significantly upregulated after transfection with hsa-let-7f-5p or hsa-miR-16-5p (Fig. 3j). In in vitro tests, BrdU incorporation in FAPs was significantly enhanced after treatment with exogenous hsa-let-7f-5p or hsa-miR-16-5p at 5 DPI and 10 DPI in injured muscles (Fig. 3k).

### TGFBR3 is the key downstream target of hsa-miR-16-5p and hsa-let-7f-5p

To identify the potential target gene of these two miRNAs, we first analyzed the transcriptome in FAPs treated by Y-PRP-exos or vehicles. GO analysis indicated that adipogenesis-related pathways were significantly downregulated in the Y-PRP-exos treated group, consistent with our previous findings (Supplementary Fig. 3a). Then, a crossover transcriptome and bioinformatic analysis indicated that only TGFBR3 was present in both lists (Supplementary Fig. 3b). To further confirm TGFBR3 as the most important candidate, we also detected other predicted genes that were reported to be associated with proliferation or anti-adipogenesis, including *Sox6*, *Runx1t1* and *Tgfbr3*. The results demonstrated that *TGFBR3* mRNA expression was significantly upregulated during FAP adipogenesis, but was significantly downregulated after treatment with Y-PRP-exos (Supplementary Fig. 3c, d). When isolating FAPs from glycerol-injured muscles at each time point, the mRNA level of TGFBR3 was gradually upregulated after injury and reached the peak at 14 DPI, which was consistent with the trajectory of FI (Supplementary Fig. 3e). These results imply that TGFBR3 may be involved in the adipogenesis of FAPs and could be a candidate target gene of hsa-let-7f-5p and hsa-miR-16-5p.

**Fig. 4:**
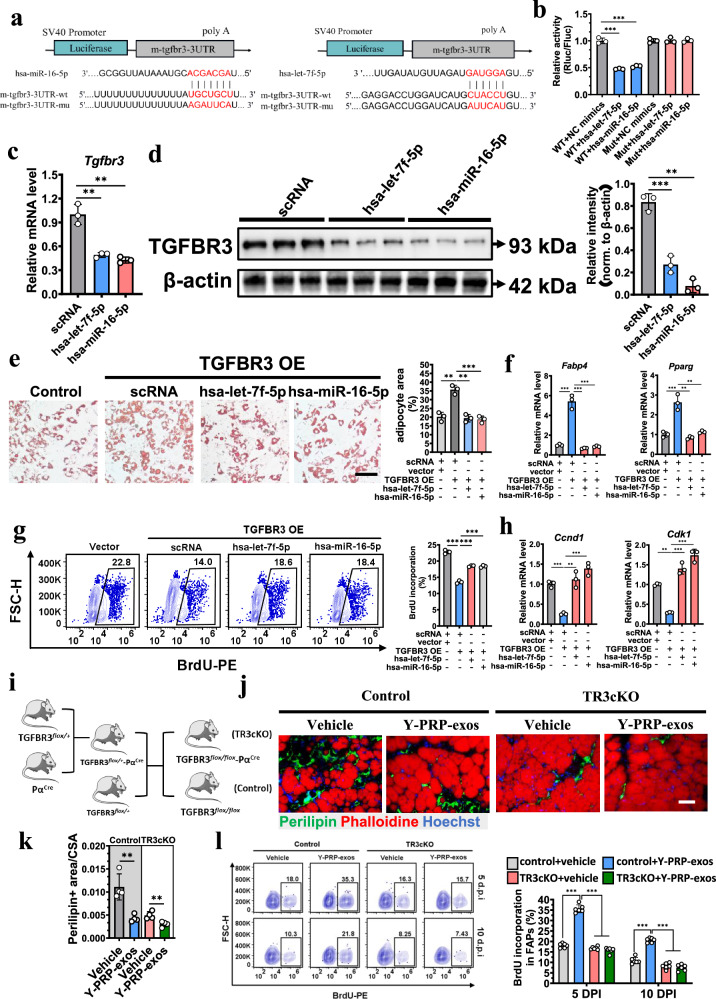
TGFBR3 is the target gene of hsa-let-7f-5p and hsa-miR-16-5p. **a** Predicted target sites of hsa-miR-16-5p or hsa-let-7f-5p binding to the 3′UTR of TGFBR3. **b** Dual-luciferase reporter system detecting 3′UTR of hsa-let-7f-5p and hsa-miR-16-5p binding to the wild type or mutated promoter region of TGFBR3. *n* = 3 per condition. **c** FAPs were cultured in adipogenic induction medium after transfection with hsa-let-7f-5p or hsa-miR-16-5p. The mRNA level of TGFBR3 was detected by RT–PCR. *n* = 3 per condition. **d** Western blots analysis showing the protein level of TGFBR3 in FAPs after transfection with hsa-let-7f-5p and hsa-miR-16-5p (left) and quantification of relative intensity of TGFBR3 compared to β-actin (right). *n* = 3 per condition. **e** Representative images of adipogenic induction of FAPs after transfection with TGFBR3 overexpression vectors with or without hsa-let-7f-5p or hsa-miR-16-5p (scale bar, 50 μm) (left) and quantification of the area of adipocytes in each group (right). *n* = 3 per condition. **f** RT–PCR analysis detecting the mRNA expression of adipogenic biomarkers in FAPs cultured in adipogenic induction medium after overexpression of TGFBR3 with or without transfection of hsa-let-7f-5p or hsa-miR-16-5p. *n* = 3 per condition. **g** Flow cytometry plots (left) and quantification (right) of BrdU incorporation in FAPs after overexpressing TGFBR3 with or without transfection of hsa-let-7f-5p or hsa-miR-16-5p. *n* = 3 per condition. **h** RT–PCR analysis detecting the mRNA expression of proliferative biomarkers in FAPs cultured in adipogenic induction medium after overexpression of TGFBR3 with or without transfection of hsa-let-7f-5p or hsa-miR-16-5p. *n* = 3 per condition. **i** Schematics of generating TR3cKO mice. **j**, **k** Representative images (**j**) (scale bar, 100 μm) and quantification (k) of FI in muscles collected from TR3cKO mice or control mice after administration of Y-PRP-exos or not. (**l**), Flow cytometry plots (right) and quantification (left) of BrdU incorporation in FAPs isolated from glycerol-injured muscles at 5 DPI and 10 DPI in TR3cKO mice or control mice after administration of Y-PRP-exos or not. *n* = 6 per condition. OE overexpression.

The dual-luciferase reporter assay confirmed that hsa-miR-16-5p and hsa-let-7f-5p can bind to the 3′UTR site of TGFBR3 (Fig. 4a, b). The mRNA and protein levels of TGFBR3 were downregulated significantly after transfection of hsa-miR-16-5p and hsa-let-7f-5p (Fig. 4c, d), indicating that TGFBR3 is the target gene of these two miRNAs.

Next, we explored the role of TGFBR3 in adipogenesis and proliferation of FAPs. As expected, overexpression of TGFBR3 induced the adipogenesis of FAPs and this was reversed after transfection of hsa-miR-16-5p and hsa-let-7f-5p (Fig. 4e, f and Supplementary Fig. 3f). Furthermore, BrdU incorporation was inhibited in the TGFBR3-overexpression group; however, transfection of hsa-miR-16-5p and hsa-let-7f-5p attenuated the effects of TGFBR3 on the proliferation of FAPs in vitro (Fig. 4g,h).

To further explore the role of TGFBR3 in regulating FAP fate in vivo, we generated mice in which TGFBR3 was specifically knocked out in FAPs (thereafter named TR3cKO) (Fig. 4i and Supplementary Fig. 3g). Interestingly, we found knocked out TGFBR3 in FAPs significantly prevented FI and the function of Y-PRP-exos on FI was attenuated (Fig. 4j, k). Moreover, the proliferation of was FAPs also significantly upregulated in TR3cKO mice and administration of Y-PRP-exos showed much less effect on the proliferation of FAPs compared to that in control mice (Fig. 4l). Together, these results indicate that TGFBR3 was the key downstream gene of Y-PRP-exos as well as hsa-miR-16-5p and hsa-let-7f-5p to regulate the adipogenesis and proliferation of FAPs.

### TGFBR3 regulates the fate of FAPs through inducing the degradation of KRT10

**Fig. 5:**
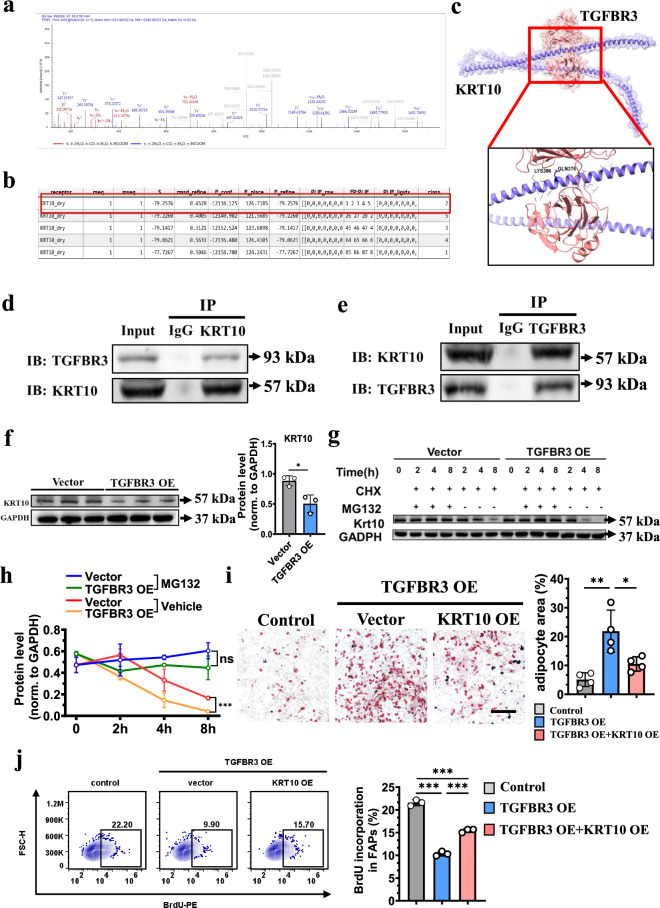
TGFBR3 regulates the cellular fate of FAPs through its interaction with KRT10. **a**, **b** LC–MS analysis (**a**) detecting the candidates that bind to TGFBR3 with different affinity (**b**) in FAPs. **c** A protein schematic showing the predicted binding sites (top) and an enlarged image (bottom). **d** Representative immunoblots (IBs) showing TGFBR3 pulled down by KRT10 in FAPs. The input TGFBR3 was used as the control. *n* = 3 per condition. **e** Representative IBs showing KRT10 pulled down by TGFBR3 in FAPs. The input KRT10 was used as the control. *n* = 3 per condition. **f** Western blot analysis detecting the protein level of KRT10 in FAPs overexpressing TGFBR3 or not (left) and quantification of the level of KRT10 normalized to the level of GAPDH (right). *n* = 3 per condition. **g**, **h** FAPs were treated by CHX with or without MG132 after overexpressing of TGFBR3 or not. Western blot analysis of the protein level of KRT10 at each time point in these conditions (**g**) and quantification of the protein level of KRT10 after normalization to the protein level of GAPDH (**h**). *n* = 3 per condition. **i** Representative images of Oil Red O staining showing the adipogenesis of FAPs after overexpression of TGFBR3 with or without overexpression of KRT10 in vitro (left) and quantification of the percentage of adipocytes area (right). *n* = 4 per condition. **j** Flow cytometry plots (left) and quantification (right) of BrdU incorporation in FAPs overexpressing of TGFBR3 with or without overexpression of KRT10. *n* = 3 per condition.

The downstream of TGFBR3 to regulate adipogenesis and proliferation was still unclear, therefore we screened the potential targets of TGFBR3 through using co-immunoprecipitation (CoIP)/liquid chromatography–mass spectrometry (LC–MS). The results showed that KRT10 was the most highly enriched and it was predicted to bind to TGFBR3 (Fig. 5a–c). CoIP confirmed the interaction between TGFBR3 and KRT10 (Fig. 5d, e). To detect how TGFBR3 regulated the protein expression of KRT10, TGFBR3 was overexpressed in FAPs. Western blot assay indicated that the protein level of KRT10 was significantly downregulated in the overexpression group compared to the control group (Fig. 5f), indicating that the protein expression of KRT10 could be inhibited by TGFBR3. In addition, the protein level of KRT10 could be preserved after pretreatment with cyclohexane (CHX) and MG132 (Fig. 5g, h), indicating KRT10 was degraded by TGBFR3 in an ubiquitination-dependent manner. We further confirmed that the function of TGFBR3 on adipogenesis and proliferation of FAPs can be regulated by KRT10. As expected, overexpression of KRT10 reversed the effects of TGFBR3 on both adipogenesis and proliferation of FAPs (Fig. 5i, j).

### TGFBR3–KRT10 axis regulates the fate of FAPs through regulating the ERK–PPARγ pathway

It is unclear how TGFBR3–KRT10 regulates adipogenesis or proliferation, therefore transcriptomics was performed to detect the DEGs in FAPs overexpressing TGFBR3 or not. In total, 826 genes were significantly upregulated and 228 genes were downregulated (Supplementary Fig. 4a). GO analysis indicated that inflammation-related pathways, ECM remodeling pathways and cell cycle pathways were significantly altered (Supplementary Fig. 4b). Specifically, apoptotic pathways and adipogenesis pathways were upregulated significantly in the TGFBR3-overexpression group compared to the control group (Supplementary Fig. 4c). Some apoptosis-related genes, such as *Mdm12, Mdm4, Il33, Casp12*, were significantly changed in these two groups (Supplementary Fig. 4d). Furthermore, GSEA confirmed that the fatty acid biosynthetic process pathway was activated and the cell growth pathway was inhibited in FAPs overexpressing TGFBR3, which are closely associated with adipogenesis and proliferation, respectively (Supplementary Fig. 4e,f).

**Fig. 6:**
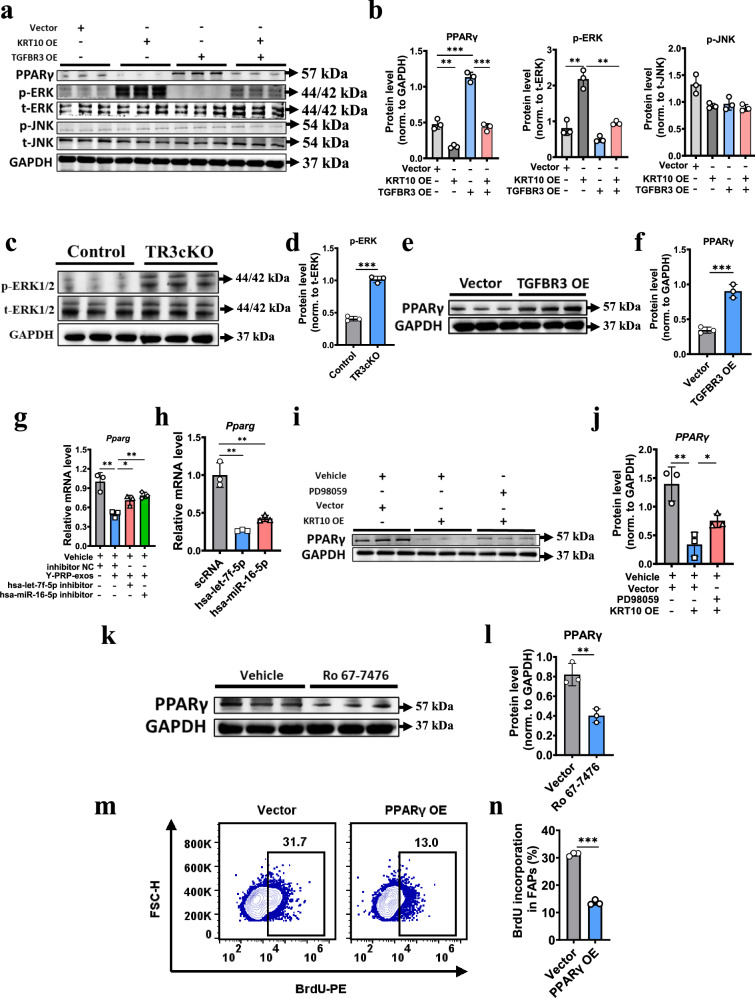
TGFBR3–KRT10 axis regulates the fate of FAPs through the ERK–PPARγ pathway. **a**, **b** Western blot analysis detecting the protein levels of PPARγ, phosphorylation of ERK and total ERK, phosphorylation of JNK and total JNK in FAPs overexpressing TGFBR3 with or without the overexpression of KRT10 (**a**) and quantification of relative protein levels of these proteins (normalized to GAPDH) (**b**). *n* = 3 per condition. **c**, **d** Western blot analysis detecting the protein levels of phosphorylation of ERK and total ERK in FAPs isolated from TR3cKO mice or control mice after glycerol injury at 7 DPI (**c**) and quantification of phosphorylated ERK normalized to total ERK (**d**). *n* = 3 per condition. **e**, **f** Western blot analysis detecting the protein levels of PPARγ in FAPs overexpressing TGFBR3 or not after induced adipogenesis (**e**) and quantification of PPARγ normalized to GAPDH (**f**). *n* = 3 per condition. **g** RT–PCR analysis showing the mRNA level of PPARγ in FAPs treated by Y-PRP-exos or not after transfection of hsa-miR-16-5p inhibitors or hsa-let-7f-5p inhibitors. *n* = 3 per condition. **h** RT–PCR analysis showing the mRNA level of PPARγ in FAPs after transfection of hsa-miR-16-5p or hsa-let-7f-5p. *n* = 3 per condition. **i**, **j** Western blot analysis detecting the protein levels of PPARγ in FAPs overexpressing KRT10 or not after treatment by an ERK antagonist during adipogenesis (**i**) and quantification of PPARγ normalized to GAPDH (**j**). *n* = 3 per condition. **k**, **l** Western blot analysis detecting the protein levels of PPARγ in FAPs treated by an ERK agonist during adipogenesis (**k**) and quantification of PPARγ normalized to GAPDH (**l**). *n* = 3 per condition. **m**, **n** Flow cytometry plots (**m**) and quantification (**n**) of BrdU incorporation in FAPs after transection of PPARγ overexpressing plasmids in vitro. *n* = 3 per condition.

Interestingly, we noticed that the ‘ERK1 and ERK2 cascade’ pathway was downregulated in the TGFBR3-overexpression group (Supplementary Fig. 4c). Consistently, the ‘MAPK signaling pathway’, which includes the ERK pathway, was upregulated in the Y-PRP-exos-treated group compared to the control group (Supplementary Fig. 3a). Numerous studies have reported that the ERK pathway regulates adipogenesis-related pathways, including fatty acid uptake and lipid droplet storage, and regulates cell proliferation and apoptosis^24–28^. These ERK-associated pathways were also significantly altered in FAPs treated by Y-PRP-exos or overexpression of TGFBR3 in this study. Thus, on the basis of the bioinformatic analysis, we hypothesized that the ERK pathway could be upstream of adipogenesis of FAPs regulated by the TGFBR3–KRT10 axis. Western blot analysis indicated that overexpression of KRT10 promoted the phosphorylation of ERK, but not JNK, and this can be reversed by the overexpression of TGFBR3 (Fig. 6a,b). Consistently, the activation of ERK was also upregulated in primary FAPs isolated from TR3cKO mice compared to in control mice (Fig. 6c,d). These data demonstrate that the TGFBR3–KRT10 axis can regulate the activation of the ERK pathway.

Next, we explored downstream components of the ERK pathway involved in regulating FAP fate. Transcriptomics showed that the ‘PPAR signaling pathway’ was downregulated in the PRP-exos-treated group compared to the control group (Supplementary Fig. 3a). PPARγ is an adipogenic biomarker and it has been reported to inhibit proliferation. Thus, we determined the protein level of PPARγ under TGFBR3 or KRT10 overexpression conditions. As expected, the protein level of PPARγ was downregulated in the KRT10-overexpression group and this was reversed by the simultaneous overexpression of TGFBR3 (Fig. 6a, b). We also observed that the expression of PPARγ was significantly upregulated in FAPs overexpressing TGFBR3 compared to the control group (Fig. 6e, f). Notably, the mRNA level of PPARγ was significantly inhibited by treatment of Y-PRP-exos and this could be reversed after transfection with an hsa-let-7f-5p or hsa-miR-16-5p inhibitor (Fig. 6g). Moreover, direct transfection of hsa-let-7f-5p or hsa-miR-16-5p also downregulated the expression of PPARγ (Fig. 6h).

To investigate whether KRT10 regulates the expression of PPARγ through the ERK pathway, FAPs were treated by an ERK antagonist after transfection of KRT10 overexpressing plasmids. Western blot analysis indicated that the protein level of PPARγ was significantly downregulated by overexpression of KRT10 and this effect was reversed by adding PD98059 (Fig. 6i, j). Similarly, the protein level of PPARγ was significantly upregulated in FAPs after treatment with an ERK agonist (Fig. 6k, l). Finally, the proliferation of FAPs was downregulated by the overexpression of PPARγ (Fig. 6m, n). Together, these data indicate that PPARγ was regulated by the TGFBR3–KRT10–ERK pathway and it inhibited the proliferation of FAPs.

### Y-PRP-exos enhances the proregenerative capacity of FAPs through upregulation of IGFBP6

**Fig. 7:**
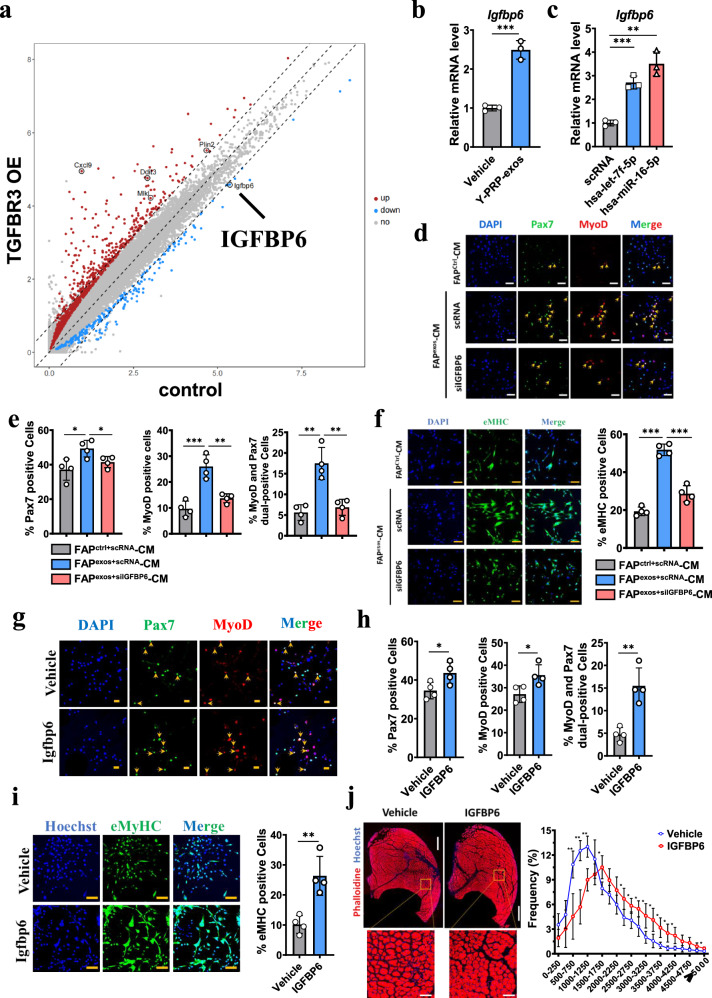
Y-PRP-exos promote the proregeneration supporting capacity of FAPs through partial upregulation of IGFBP6. **a** A scatter plot of DEGs in FAPs treated by overexpression of TGFBR3 or not. **b** RT–PCR analysis detecting the mRNA level of IGFBP6 in FAPs treated by Y-PRP-exos or not. *n* = 3 per condition. **c** RT–PCR analysis detecting the mRNA level of IGFBP6 in FAPs after transfection of hsa-miR-16-5p or hsa-let-7f-5p. *n* = 3 per condition. **d**, **e** Representative images of immunofluorescence analysis detecting the expression of Pax7 and MyoD in MuSCs treated by CM collected from FAPs treated by Y-PRP-exos with or without transfection of IGFBP6 siRNA (**d**) and quantification of the percentage of Pax7-positive cells, MyoD-positive cells or dual-positive cells (**e**). Scale bar, 50 μm. *n* = 4 per condition. **f** Representative images of immunofluorescence analysis detecting the expression of eMHC in MuSCs treated by CM collected from FAPs treated by Y-PRP-exos with or without transfection of IGFBP6 siRNA (left) and quantification of the percentage of Pax7-positive cells, MyoD-positive cells or dual-positive cells (right). Scale bar, 50 μm. *n* = 4 per condition. **g**, **h** Representative images of immunofluorescence analysis detecting the expression of Pax7 and MyoD in MuSCs treated by recombinant IGFBP6 protein or not (**g**) and quantification of the percentage of Pax7-positive cells, MyoD-positive cells or dual-positive cells (**h**). Scale bar, 50 μm. *n* = 4 per condition. **i** Representative images of immunofluorescence analysis detecting the expression of eMHC in MuSCs treated by recombinant IGFBP6 protein or not (left) and quantification of the percentage of eMHC positive cells (right). Scale bar, 50 μm. *n* = 4 per condition. **j** Representative images of myofibers in glycerol-injured muscle at 14 DPI after treatment by IGFBP6 or vehicle in whole sections (left, top) and enlarged images (left, bottom) and quantification of the distribution of CSA of myofibers (left). Scale bar, 500 μm (whole section) and 100 μm (enlarged image). *n* = 4 per condition. CSA cross sectional area.

To investigate whether Y-PRP-exos could promote the regenerative supporting capacity of FAPs to stimulate the activation of MuSCs, transcriptomics was performed to find the key regulator of tissue regeneration. We found IGFBP6 was significantly downregulated and CXCL9 was significantly upregulated in FAPs that overexpressed TGFBR3 compared to the control group (Fig. 7a). Since it was reported that CXCL9 negatively regulates and IGFBP6 positively regulates muscle regeneration, we thus hypothesized that IGFBP6 could be a key regulator for FAPs treated by Y-PRP-exos. We first confirmed that treatment of PRP-exos or transfection with hsa-let-7f-5p or hsa-miR-16-5p could significantly upregulate the mRNA level of IGFBP6 in FAPs (Fig. 7b, c). Then, conditional medium (CM) collected from FAPs transfected with IGFBP6 siRNA (thereafter named siIGFBP6) or scRNA were used to treat MuSCs. The results showed that CM collected from FAPs transfected with scRNA promoted Pax7-positive cells, MyoD-positive cells or dual-positive cells (Fig. 7d, e). However, the knocking down of IGFBP6 reversed the effects of FAP CM on the activation of MuSCs (Fig. 7d, e). Consistently, eMHC expression in MuSCs was also upregulated in CM collected from FAP transfected with scRNA but was reversed by CM collected from FAP transfected with siIGFBP6 (Fig. 7f). When treated with the recombinant IGFBP6 protein, we found that the activation of MuSCs was also upregulated in the IGFBP6-treated group compared to the control group (Fig. 7g-i). Furthermore, IGFBP6 can stimulate the proliferation of MuSCs (Supplementary Fig. 5a–c). The average area of myofibers was also significantly larger in the IGFBP6-treated group compared to the control group (Fig. 7j). Together, these results indicate that Y-PRP-exos or hsa-let-7f-5p and hsa-miR-16-5p facilitate muscle regeneration through upregulating IGFBP6.

## Discussion

The role of FAPs exerts a ‘double-edged sword’ effect. In a degenerative microenvironment, FAPs can promote the muscle degeneration, and accumulation of FAPs could lead to uncontrolled fibrosis and FI, thus impairing muscle regeneration and quality. On the contrary, in a regenerative microenvironment, FAPs could support muscle regeneration through secreting abundant proregenerative cytokines^13,15,21,22,29–31^. The transient proliferation of FAPs is essential to muscle regeneration^32,33^. Here, we found that Y-PRP-exos can promote the viability of FAPs and enhance the production of IGFBP6 while inhibiting its adipogenesis, demonstrating that Y-PRP-exos as well as its miRNA cargo can establish a proregenerative microenvironment in injured muscle through inducing the regeneration-supporting capacity of FAPs.

TGFBR3 is an important TGFβ receptor. Several studies have shown that TGFBR3 is a tumor suppressor gene that inhibits the proliferation or induces the apoptosis of cancer cells, but other studies have also found that TGFBR3 may promote proliferation in some circumstances^34–37^. We observed that TGFBR3 played a suppressor role in the proliferation of FAPs. In addition, TGFBR3 was reported to promote adipogenesis in previous studies^38^, and our findings also found that it was an important target for regulating adipogenesis of FAPs. Thus, we demonstrated TGFBR3 as an important regulator to determine the cellular fate of FAPs in acute muscle injury. TGFBR3 belongs to the transforming growth factor receptor family, with the Smad pathway and PI3K pathway commonly reported as its downstream targets^39,40^. In our study, we found that the activation of the ERK pathway was responsible for mediating the function of TGFBR3 on the adipogenesis and proliferation of FAPs. The ERK pathway exerted an adipogenesis-inhibition effect, in agreement with previous studies^24,41^. Some studies have demonstrated that the ERK pathway is closely associated with the phosphorylation of PPARγ; however, here, we found that both the mRNA level and the protein level of PPARγ could be regulated by the ERK pathway.

KRT10 was identified as a new target protein of TGFBR3. KRT10 is predominantly involved in inflammation and has not been reported to be associated with adipogenesis in previous studies^42^. However, here we explored the role of KRT10 in inhibiting the adipogenesis of FAPs through activation of ERK. In addition, although KRT10 was found to inhibit the proliferation of some types of cancer cells^43^, we found that overexpression of KRT10 preserved the viability of FAPs, indicating that the KRT10 has distinct roles in cell proliferation based on the cell type and local microenvironment.

Aging is a critical factor modulating the internal environment, which may impact the contents of exosomes^44^. One previous study also demonstrated that blood products from young donors can reverse aging^45^. Consistently, in our study, we proved that the enrichment of bioactive components in PRP-exos collected from old donors was markedly reduced compared to that in young donors, indicating that blood products can be significantly remodeled by aging. This indicates that aging should be considered as a potential confounder that impacts the therapeutic effects of blood products.

Specifically older people often suffer from musculoskeletal diseases, such as RCTs. Since PRP can currently only be used in the manner of autologous transfusion, we suggest that the dosage of PRP should be increased when treating elderly people.

Although RNAs, DNAs and metabolites are enriched in exosomes, we found that miRNAs showed a superior effect on regulating the cellular fate of FAPs. hsa-let-7f-5p and hsa-miR-16-5p were the two top enriched miRNAs in PRP-exos, regardless of age. Although hsa-let-7f-5p and hsa-miR-16-5p were found to participate in tumorigenesis or mental diseases, neither of these miRNAs were reported to be associated with adipogenesis of mesenchymal stem cells^46–48^. We here first demonstrated that these two miRNAs were critical components for PRP-exo-mediated inhibition of adipogenesis and promote proliferation by targeting TGFBR3.

We determined that PRP-exos collected from young donors facilitated muscle regeneration through regulating the cellular fate of FAPs. hsa-miR-16-5p and hsa-let-7f-5p were two key components that mediate Y-PRP-exos in regulating FAPs and FI in injured muscles. Importantly, we identified that TGFBR3 is a critical regulator for adipogenesis and proliferation of FAPs and that the TGFBR3–KRT10–ERK axis is an important downstream component of Y-PRP-exos or these two miRNAs. Another key finding in our study is that aging is a critical confounder that affects the enrichment of the miRNA cargo in PRP-exos and impairs the therapeutic effects of PRP-exos on muscle injury. This study clarified the underlying mechanisms of PRP-exos regulation of FI in injured muscles as well as muscle regeneration and explored TGFBR3 as a new therapeutic target to treat FI. Moreover, since TGFBR3 was found to be an important inhibitor for tumorigenesis, especially in elder people, the broad inhibition of TGFBR3 may raise the concern for susceptibility to cancer^40,45^. Although we explored that administration of PRP-exos showed little toxicity to major organs in the short term, the long-term side effects were not analyzed in this study. Considering this, a delivery system containing hsa-miR-16-5p and hsa-let-7f-5p specifically targeting FAPs could be a promising way to reduce the risk of developing tumors.

## Supplementary information

Supplementary Information

## Data Availability

The RNA-seq data from this study have been submitted to the NCBI Gene Expression Omnibus (GEO) (https://www.ncbi.nlm.nih.gov/geo) under accession numbers GEO: GSE283671 and GSE284069. All software used was freely or commercially available.
