# Supplementary material for: Platelet-rich plasma-derived exosomes establishing a muscular proregenerative microenvironment through enhancing the viability of fibro-adipogenic progenitors
Source: Exp Mol Med. 2025 Dec 25;57(12):2957–71. doi: 10.1038/s12276-025-01606-x (PMC12800062; doi:10.1038/s12276-025-01606-x)
Supplement: Supplementary file 1 — Supplementary Information [file 12276_2025_1606_MOESM1_ESM.pdf]

Supplementary Figures

Supplementary Fig 1.

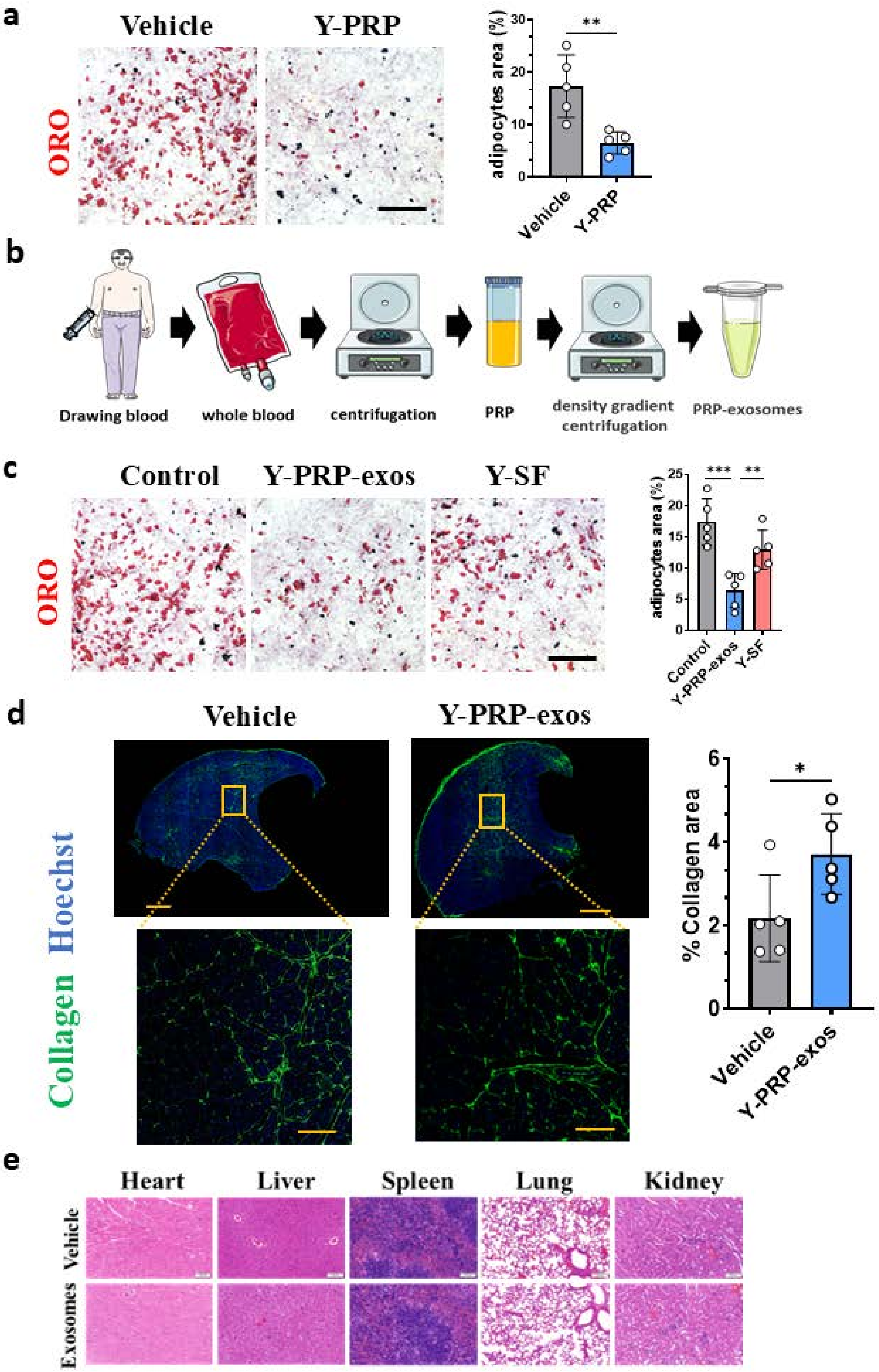

Supplementary Fig. 1. PRP-exos is the key regulator contained in PRP to inhibit the adipogenesis of FAPs.

(a) Representative images of adipogenic induction of FAPs after treatment of PRP or vehicle detected by Oil red O (Scale bar = 50μm) (left) and quantification of the area of adipocytes in each group (right). n=5 per condition.

(b) Schematics of isolating exosomes from PRP.

(c) Representative images of adipogenic induction of FAPs after treatment of PRP or soluble factor (SF) detected by Oil red O (Scale bar = 50μm) (left) and quantification of the area of adipocytes in each group (right). n=5 per condition.

(d) Representative images of immunofluorescence detecting the expression of Collagen I in glycerol-injured muscles at 14 DPI treated by Y-PRP-exos or vehicle, respectively. The collagen I expression in whole sections (left, upper) (scale bar = 500μm) or enlarged images (left, lower) (scale bar = 50μm) was evaluated and quantification of the percentage of fibrosis area was detected (right). n=5 per condition.

(e) Representative images of hematoxylin-eosin staining evaluating the injury signs of major organs after treatment of Y-PRP-exos. Heart, Lung, Liver, kidney and spleen were harvested from mice after treatment of Y-PRP-exos or PBS. The tissue morphology of these organs was detected through using hematoxylin-eosin staining, respectively (scale bar = 100μm). n=3 per condition.

Supplementary Fig 2.

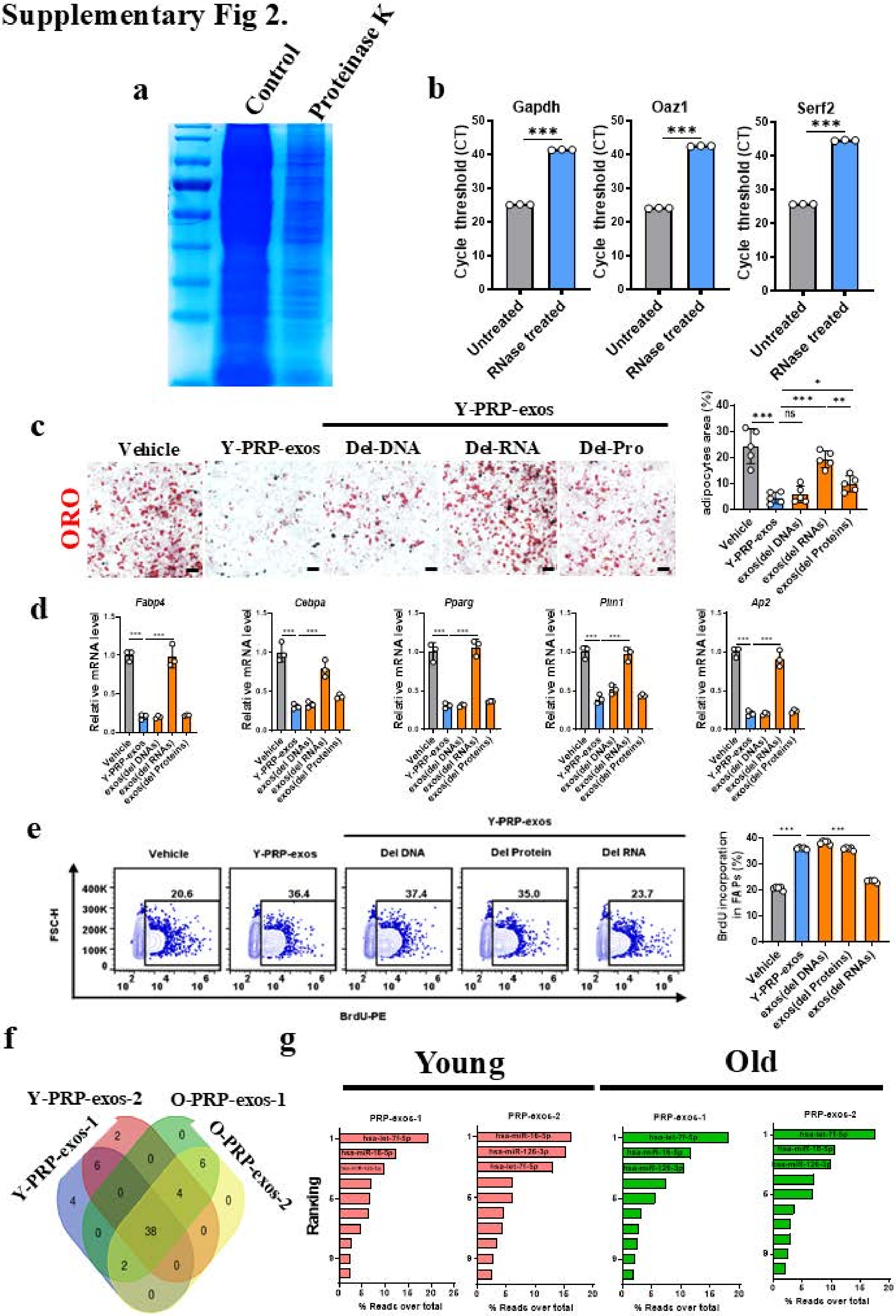

Supplementary Fig. 2. RNAs were the dominant bioactive factor to mediate the effects of Y-PRP-exos on adipogenesis and proliferation of FAPs.

(a) Detecting the efficiency of depletion of proteins.

(b) Real-time PCR detecting the efficiency of depletion of RNAs in exosomes treated by RNase.

(c) Representative images of adipogenic induction of FAPs after treatment of Y-PRP-exos with or without depletion of proteins, DNAs or RNAs, respectively (Scale bar =50μm) (left) and quantification of the area of adipocytes in each group (right). n=5 per condition.

(d) Real-time PCR analysis detecting the expression of adipogenic biomarkers in FAPs after treatment of Y-PRP-exos with or without depletion of proteins, DNAs or RNAs, respectively. n=3 per condition.

(e) Flow cytometry plots (left) and quantification (right) of BrdU incorporation in FAPs after treatment of Y-PRP-exos with or without depletion of proteins, DNAs or RNAs, respectively. n=3 per condition.

(f) Venn diagram showing the common miRNAs in FAPs treated by Y-PRP-exos or O-PRP-exos.

(g) The top 3 enriched miRNAs in Y-PRP-exos or O-PRP-exos detecting by miRNA-Seq.

Supplementary Fig 3.

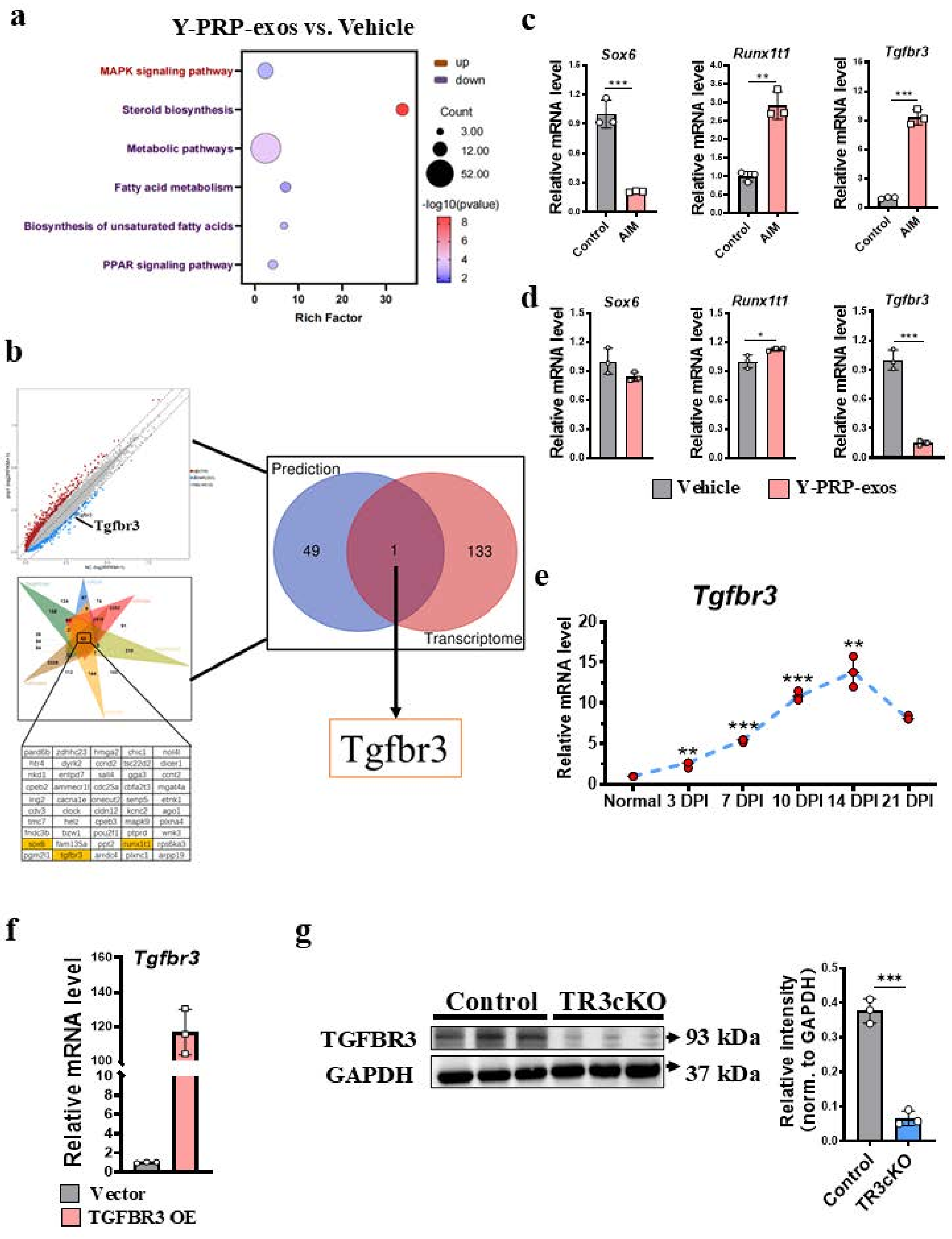

Supplementary Fig. 3. TGFBR3 is regulated by Y-PRP-exos.

(a) GO analysis showed the significantly changed pathways in FAPs overexpressing TGFBR3 or not detected by RNA-Seq.

(b) The differentially expressed genes in FAPs overexpressing TGFBR3 or not detected by RNA-Seq (left, upper) and bioinformatic analysis of prediction of the target genes of hsa-let-7f-5p and hsa-miR-16-5p (left, lower). The co-expressed genes were presented (right).

(c) Real-time PCR analysis detecting the mRNA expression of Sox6, Runx1t1 and TGFBR3 in FAPs cultured in adipogenic induction medium. n=3 per condition.

(d) Real-time PCR analysis detecting the mRNA expression of Sox6, Runx1t1 and TGFBR3 in FAPs cultured in adipogenic induction medium treated by Y-PRP-exos or not. n=3 per condition.

(e) Real-time PCR analysis detecting the expression of TGFBR3 in FAPs isolated from glycerol-injured muscles at each timepoint. n=3 per condition.

(f) Real-time PCR analysis detecting the expression of TGFBR3 in FAPs after transfection of TGFBR3 overexpressing plasmids or vectors. n=3 per condition.

(g) Western blot analysis showing the expression of TGFBR3 in FAPs isolated from TR3cKO mice or control mice (left) and quantification of the relative protein level of TGFBR3 after normalized to GAPDH (right). n=3 per condition.

Supplementary Fig 4.

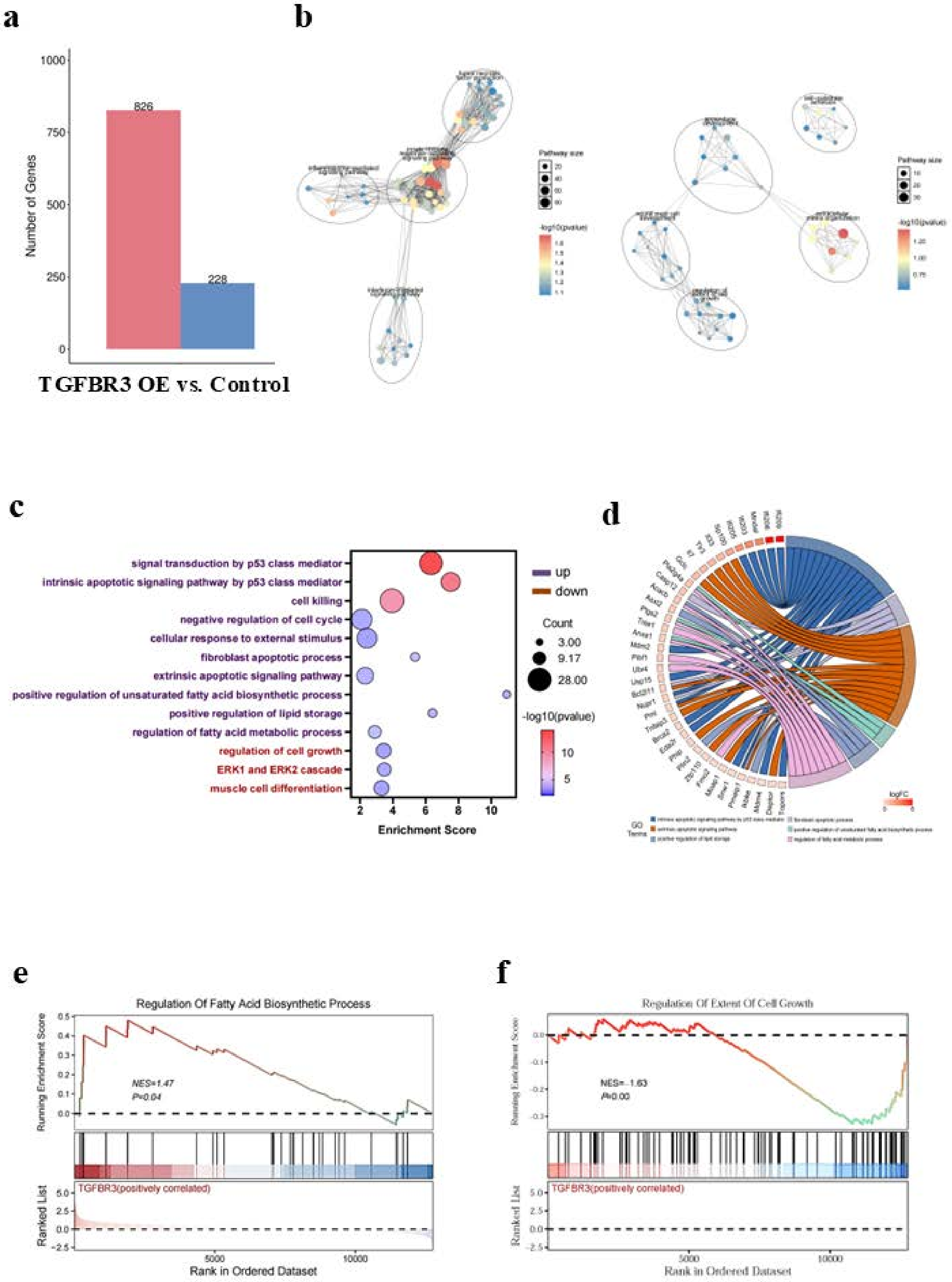

Supplementary Fig. 4. Overexpressing TGFBR3 impacting the adipogenesis and proliferation of FAPs.

(a) The differentially upregulated or downregulated genes in FAPs after overexpressing TGFBR3 or not.

(b) The network of differentially expressed pathways of GO analysis in FAPs after overexpressing TGFBR3 or not.

(c) Bubble diagram of GO analysis showing the differentially expressed pathways related to apoptosis or adipogenesis.

(d) Chordal graph presenting the common differentially expressed genes in apoptosis-related pathways or adipogenesis-related pathways.

(e) GSEA analysis showed “Regulation of fatty acid biosynthetic process” pathway differentially expressed in FAPs overexpressing TGFBR3 or not.

(f) GSEA analysis showed “Regulation of extent of cell growth” pathway differentially expressed in FAPs overexpressing TGFBR3 or not.

Supplementary Fig 5.

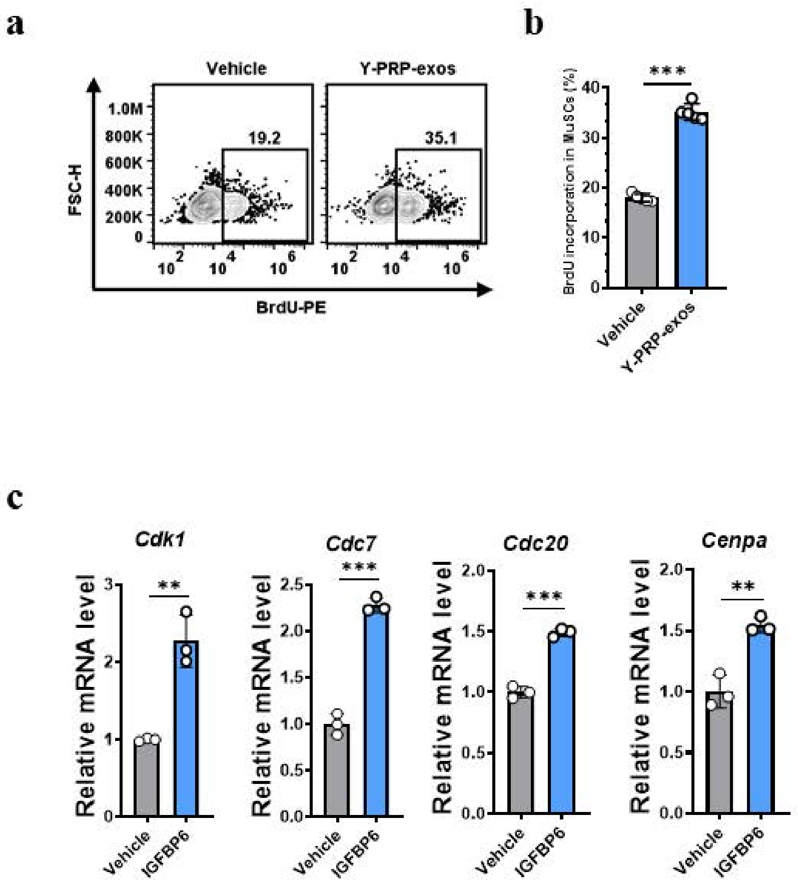

Supplementary Fig. 5. IGFBP3 promoting the proliferation of MuSCs.

(a-b) Flow cytometry plots (a) and quantification (b) of BrdU incorporation in MuSCs after treatment of IGFBP6 or not. n=3 per condition.

(c) Real-time PCR analysis detecting the expression of cell cycle biomarkers in MuSCs treated by IGFBP6 or not. n=3 per condition.

Supplementary Table 1. Key resources table

REAGENT or RESOURCE	SOURCE	IDENTIFIER
Antibodies		
PE anti-mouse CD140a Antibody	Biolegend	Clone#:APA5; Cat#135906; RRID: AB_1953269
Alexa Fluor® 488 anti-mouse CD31 Antibody	Biolegend	Clone#:390; Cat#102414; RRID:AB_493408
Alexa Fluor® 488 anti-mouse CD45 Antibody	Biolegend	Clone#:30-F11; Cat#103122; RRID: AB_493531
APC/Cyanine7 anti-mouse Ly-6A/E (Sca-1) Antibody	Biolegend	Clone#:D7; Cat#108126; RRID: AB_10645327
PE anti-BrdU Antibody	Biolegend	Clone#:3D4; Cat#364116; RRID: AB_2814317
FITC anti-BrdU Antibody	Biolegend	Clone#:3D4; Cat#364104; RRID: AB_2564481
COL1A1 Antibody	SANTA CRUZ	Clone#:3G3; Cat#sc-293182; RRID: AB_2797597
Anti-Perilipin-1 antibody	ABCAM	Cat#ab61682; RRID:AB_944751
Anti-Ki67 antibody	ABCAM	Clone#:SP6; Cat#ab16667; RRID: AB_302459
Mouse PDGF R alpha Antibody	R&D	Cat#AF1062; RRID: AB_2236897
Myosin Heavy Chain Recombinant Rabbit Monoclonal Antibody	Invitrogen	Clone#:6V0G1; Cat#MA5-35613; RRID: AB_2849513
TRITC Phalloidin	Yeason	Cat#40734ES80;
iFluor™ 647 phalloidin	Yeason	Cat#40762ES75;
KRT10 Antibody	SANTA CRUZ	Clone#:DE-K10; Cat#sc-52318; RRID: AB_629836
beta Actin Antibody	Affinity Biosciences	Cat#AF7018; RRID: AB_2839420
GAPDH Antibody	Affinity Biosciences	Cat#AF7021; RRID: AB_2839421

TGF-β Receptor III Antibody	Cell Signaling Technology	Cat#2519; RRID: AB_390707
PPAR gamma Antibody	Affinity Biosciences	Cat#AF6284; RRID: AB_2835135
ERK1/2 Antibody	Affinity Biosciences	Cat#AF0155; RRID: AB_2833336
Phospho-ERK1/2 (Thr202/Tyr204) Antibody	Affinity Biosciences	Cat#AF1015; RRID: AB_2834432
JNK1/2/3 Antibody	Affinity Biosciences	Cat#AF6318; RRID: AB_2835177
Phospho-JNK1/2/3 (Thr183+Tyr185) Antibody	Affinity Biosciences	Cat#DF3318; RRID: AB_2834737
Anti-MyoD1 Antibody	ABCAM	Cat#ab133627; RRID: AB_2890928
PAX7 Antibody	DSHB	RRID:AB_528428
Alexa Fluor® 488 AffiniPure Donkey Anti-Chicken IgY (IgG) (H+L)	Jackson ImmunoResearch	Code: 703-545-155; RRID: AB_2340375
Alexa Fluor® 488 AffiniPure Donkey Anti-Mouse IgG (H+L)	Jackson ImmunoResearch	Code: 715-545-151; RRID: AB_2341099
Cy™3 AffiniPure Donkey Anti-Mouse IgG (H+L)	Jackson ImmunoResearch	Code: 715-165-150; RRID: AB_2340813
Alexa Fluor® 488 AffiniPure Donkey Anti-Rabbit IgG (H+L)	Jackson ImmunoResearch	Code: 711-545-152; RRID: AB_2313584
Alexa Fluor® 488 AffiniPure Donkey Anti-Goat IgG (H+L)	Jackson ImmunoResearch	Code: 705-545-003; RRID: AB_2340428
Alexa Fluor® 488 AffiniPure Donkey Anti-Rat IgG (H+L)	Jackson ImmunoResearch	Code: 712-545-150; RRID: AB_2340683
Cy™3 AffiniPure Donkey Anti-Rat IgG (H+L)	Jackson ImmunoResearch	Code: 712-165-150; RRID: AB_2340666
Cy™3 AffiniPure Donkey Anti-Rabbit IgG (H+L)	Jackson ImmunoResearch	Code: 712-165-152; RRID: AB_2307443
Cy™3 AffiniPure Donkey Anti-Goat IgG (H+L)	Jackson ImmunoResearch	Code: 705-165-003; RRID: AB_2340411
Horseradish enzyme labeled goat anti-rabbit IgG (H+L)	ZSGB-Bio	Cat#ZB-2301; RRID:AB_2747412
Horseradish enzyme labeled goat anti-mouse IgG (H+L)	ZSGB-Bio	Cat#ZB-2305; RRID:AB_2747415
Recombinant Mouse IGFBP-6	Biolegend	Cat#752404
Chemicals, peptides, and recombinant proteins		
MG-132	MedChemExpress	Cat#HY-13259
Cycloheximide	MedChemExpress	Cat#HY-12320
Hoechst 33342	Beyotime	Cat#C1026

Tamoxifen	Sigma	Cat#T5648-5G
Collagenase Type II	BioFroxx	Cat#2275GR001
Tissue TeK O.C.T.Compound	SAKURA	Cat#4583
INVI DNA RNA Transfection Reagent	Invigentech	Cat#IV1216075
Glycerol	BioFroxx	Cat#1280ML100
Critical commercial assays		
Phase-Flow™ FITC BrdU Kit	Biolegend	Cat#370704
PrimeScript™ RT Master Mix	Takara	Cat#RR036A
TB Green® Premix Ex Taq™ II	Takara	Cat#RR820A
Modified Oil Red O Stain Kit	Solarbio	Cat#G1261
Coomassie Blue Staining Solution	Beyotime	Cat#P0017B
Deposited data		
Experimental models: Organisms/strains		
C57BL/6J	HUNAN SJA LABORATORY ANIMAL CO.,LTD	N/A
Mouse:B6N.Cg-Tg(Pdgfra-cre/ERT)467Dbe/J	The Jackson Laboratory	RRID:IMSR_JAX:018280
Mouse:C57BL/6JCya-Tgfbr3^{em1flox}/Cya	Cyagen Bio- sciences Inc. (Suzhou, China)	Cat#S-CKO-08178
Oligonucleotides		
REAGENT or RESOURCE	SOURCE	IDENTIFIER
Genotyping primer: PDGFRα-CreERT Forward: TCAGCCTTAAGCTGGGACAT	The Jackson Laboratory	14633
Genotyping primer: PDGFRα-CreERT Reverse: ATGTTTAGCTGGCCCAAATG	The Jackson Laboratory	oIMR9377
Genotyping primer: TGFBR3 Forward: ATATGTGCCCTATTAAGGGGCTAAG	Cyagen Bio- sciences Inc. (Suzhou, China)	N/A
Genotyping primer: TGFBR3 Reverse: ATCTCTACAGCAGGGTGGTTTCTA	Cyagen Bio- sciences Inc. (Suzhou, China)	N/A
qPCR Primer: CDC20 Forward: GCCGAACTCCTGGCAAATCT	Primer Bank	N/A
qPCR Primer: CDC20 Reverse: TTGGGGGATAAAGCGGTCAC	Primer Bank	N/A
qPCR Primer: CDK1 Forward: GGTCCGTCGTAACCTGTTGA	Primer Bank	N/A
qPCR Primer: CDK1 Reverse: CCACACCGTAAGTACCTTCTCC	Primer Bank	N/A

qPCR Primer: CDCA3 Forward: GAGGAAGCCAAACAATCCGC	Primer Bank	N/A
qPCR Primer: CDCA3 Reverse: TGCGCTTAGAACCTGAGGAC	Primer Bank	N/A
qPCR Primer: CENPA Forward: GGCCCTTCAGGAGACATTACC	Primer Bank	N/A
qPCR Primer: CENPA Reverse: GGCACCGTGTAGCCAGTATT	Primer Bank	N/A
qPCR Primer: IGFBP6 Forward: GGTCTACAGCCCTAAGTGCG	Primer Bank	N/A
qPCR Primer: IGFBP6 Reverse: CCTTTGTAGTCTCCTCCGACG	Primer Bank	N/A
qPCR Primer: CXCL9 Forward: CTCGGACTTCACTCCAACACA	Primer Bank	N/A
qPCR Primer: CXCL9 Reverse: ATCACTAGGGTTCCTCGAACT	Primer Bank	N/A
qPCR Primer: Gapdh Forward: CCTGGAGAAACCTGCCAAGTATG	Primer Bank	N/A
qPCR Primer: Gapdh Reverse: AGAGTGGGAGTTGCTGTTGAAGTC	Primer Bank	N/A
qPCR Primer: FABP4 Forward: CGACAGGAAGGTGAAGAGCATC	Primer Bank	N/A
qPCR Primer: FABP4 Reverse: ATTCCACCAGCTTGTCACCA	Primer Bank	N/A
qPCR Primer: Perilipin Forward: GAGACTGAGGTGGCGGTCT	Primer Bank	N/A
qPCR Primer: Perilipin Reverse: CATCCTTACTCTCCACGCTGTAAC	Primer Bank	N/A
qPCR Primer: PPARγ Forward: TCGCTGATGCACTGCCTATG	Primer Bank	N/A
qPCR Primer: PPARγ Reverse: GAGAGGTCCACAGAGCTGATT	Primer Bank	N/A
qPCR Primer: C/EBPa Forward: GCGGGAACGCAACAACATC	Primer Bank	N/A
qPCR Primer: C/EBPa Reverse: GTCACTGGTCAACTCCAGCAC	Primer Bank	N/A
qPCR Primer: LEPTIN Forward: TGTCCTACTGCTCGGAACAC	Primer Bank	N/A
qPCR Primer: LEPTIN Reverse: GCTCAAATGTTTCAGGCTTTTGG	Primer Bank	N/A
qPCR Primer: CCND1 Forward: CAGCCCCAACAACTTCCTCT	Primer Bank	N/A
qPCR Primer: CCND1 Reverse: CAGGGCCTTGACCGGG	Primer Bank	N/A

qPCR Primer: CDC7 Forward: TGGGATTTGGACATATGCTGCT	Primer Bank	N/A
qPCR Primer: CDC7 Reverse: AAGAGGCTTCCACTACGCAC	Primer Bank	N/A
qPCR Primer: AP2 Forward: TACGAAGACTGCGAGGACCG	Primer Bank	N/A
qPCR Primer: AP2 Reverse: AAGTCGGCATTAGGGGTGTG	Primer Bank	N/A
qPCR Primer: LPL Forward: TACCCCCTAGACAACGTCCA	Primer Bank	N/A
qPCR Primer:LPL Reverse: CCAGCTGGATCCAAACCAGT	Primer Bank	N/A
qPCR Primer: SOX6 Forward: AAGGCAGTTGCCAATGTGAC	Primer Bank	N/A
qPCR Primer: SOX6 Reverse: CTTGCTTGGAAGACATTCTTAGTTA	Primer Bank	N/A
qPCR Primer: RUNX1T1 Forward: TGCCTTTTGTTGTGCTGGGT	Primer Bank	N/A
qPCR Primer: RUNX1T1 Reverse: CCACAGATCAATCTTTTCTCCGC	Primer Bank	N/A
qPCR Primer: TGFBR3 Forward: TGCAAGGGGGCGTGAATATC	Primer Bank	N/A
qPCR Primer: TGFBR3 Reverse: CGAGTAGCCATTGGTCTGGA	Primer Bank	N/A
siRNAs		
IGFBP6 siRNA (mus) Forward: GGAGCAGUGACUUCAUUCATT	Shanghai GenePharma Co.,Ltd	N/A
IGFBP6 siRNA (mus) Reverse: UGAAUGAAGUCACUGCUCCTT	Shanghai GenePharma Co.,Ltd	N/A
hsa-let-7f-5p(homo) mimics Forward: UGAGGUAGUAGAUUGUAUAGUU	Shanghai GenePharma Co.,Ltd	N/A
hsa-let-7f-5p(homo) mimics Reverse: CUAUACAAUCUACUACCUCAUU	Shanghai GenePharma Co.,Ltd	N/A
hsa-mir-16-5p(homo) mimics Forward: UAGCAGCACGUAAAUAUUGGCG	Shanghai GenePharma Co.,Ltd	N/A
hsa-mir-16-5p(homo) mimics Reverse: CCAAUAUUUACGUGCUGCUAUU	Shanghai GenePharma Co.,Ltd	N/A
Recombinant DNA		
Plasmid: TGFBR3	Shanghai GeneChem Co.,Ltd	N/A
Plasmid:KRT10	Shanghai GeneChem Co.,Ltd	N/A
Plasmid:PPARγ	Shanghai GeneChem Co.,Ltd	N/A

Software and algorithms		
BD FACSAria III Cell sorter	BD Biosciences	N/A
BD FACSCalibur	BD Biosciences	N/A
Bio-Rad ChemiDoc MP System	Bio-Rad	N/A
Leica SP8 confocal microscope	Leica	N/A
Nikon A1R HD25	Nikon	N/A
FlowJo v10	FlowJo	N/A
ImageJ	N/A	https://imagej.nih.gov/ij/
Prism 7 and 8	GraphPad Software, Inc	N/A
